# Supplementary material for: Two-Year Study on the Intra-Individual Dynamics of Gut Microbiota and Short-Chain Fatty Acids Profiles in Healthy Adults
Source: Microorganisms. 2024 Aug 20;12(8):1712. doi: 10.3390/microorganisms12081712 (PMC11357285; doi:10.3390/microorganisms12081712)
Supplement: Supplementary file 1 [file microorganisms-12-01712-s001.zip › microorganisms-3144091-Table S1.pdf]

Supplementary Table 1. Characteristics of study participants

ID	Sex	Age*	BMI	Number of samples
1	Female	27	16,7	10
2	Male	31	25	10
3	Female	29	22,6	9
4	Female	26	21,1	9
5	Female	30	20,5	10
6	Female	30	16,3	10
7	Female	27	23,05	10
8	Female	34	20	6
9	Female	28	19,8	5
10	Female	37	20,55	10
11	Female	36	17,9	10
12	Female	26	23,3	9
13	Female	52	24,6	10
14	Female	37	25	10
15	Male	50	25	10

* At the start of the study
