# Supplementary material for: Two-Year Study on the Intra-Individual Dynamics of Gut Microbiota and Short-Chain Fatty Acids Profiles in Healthy Adults
Source: Microorganisms. 2024 Aug 20;12(8):1712. doi: 10.3390/microorganisms12081712 (PMC11357285; doi:10.3390/microorganisms12081712)
Supplement: Supplementary file 1 [file microorganisms-12-01712-s001.zip › microorganisms-3144091-Table S2.pdf]

Supplementary Table 2. Statistics of alpha diversity indices

Participant ID	Shannon index				Observed features			
	min	max	mean±SD	SD %	min	max	mean±SD	SD %
1	5,5	6,6	6,2±0,3	4,7	213	338	276,7±44,4	16
2	5,3	6,1	5,7±0,2	4,4	154	213	176,4±20,2	11,4
3	5	6,9	5,9±0,6	10,2	192	386	289,38±66	22,8
4	6,1	6,8	6,4±0,3	4	186	390	282,4±71,9	25,5
5	5,9	6,4	6,1±0,2	2,5	185	312	244±34,4	14,1
6	5,9	6,7	6,3±0,3	4,9	145	346	271,7±54,9	20,2
7	6,2	6,9	6,5±0,3	4,4	165	333	258,33±56,8	22
8	6	6,4	6,2±0,1	2,2	167	253	221,5±31,6	14,3
9	5,9	6,8	6,3±0,4	5,6	203	286	256,8±30,3	11,8
10	6,4	6,8	6,6±0,1	1,9	234	407	302,8±54	17,8
11	5,8	6,5	6,2±0,3	4,2	144	276	215,2±44,1	20,5
12	5,5	6,3	6±0,2	3,8	174	248	221,5±26	11,7
13	5,2	6,5	5,9±0,4	6,7	160	313	226,8±50,4	22,2
14	5,8	7,2	6,6±0,4	6,4	152	385	264,4±69,9	26,4
15	5,8	6,7	6,4±0,3	4,4	165	291	233,3±33,8	14,5
