# Supplementary material for: Two-Year Study on the Intra-Individual Dynamics of Gut Microbiota and Short-Chain Fatty Acids Profiles in Healthy Adults
Source: Microorganisms. 2024 Aug 20;12(8):1712. doi: 10.3390/microorganisms12081712 (PMC11357285; doi:10.3390/microorganisms12081712)
Supplement: Supplementary file 1 [file microorganisms-12-01712-s001.zip › microorganisms-3144091-Table S3.pdf]

Supplementary Table 3. Amounts of short-chain fatty acids in fecal samples from healthy volunteers

Participants	C2 (mg/g) (acetic)	C3 (mg/g) (propionic)	iC4 (mg/g) (isobutyric)	C4 (mg/g) (butyric)	iC5 (mg/g) (isovaleric)	C5 (mg/g) (valeric)	iC6 (mg/g) (isocaproic)	C6 (mg/g) (caproic)
1	1,2±0,3	0,3±0,1	0,07±0,02	0,2±0,1	0,17±0,06	0,10±0,02	0,003±0,003	0,06±0,02
2	1,7±0,7	0,7±0,2	0,08±0,03	0,6±0,3	0,19±0,08	0,14±0,04	0,004±0,003	0,02±0,02
3	1,3±0,7	0,4±0,2	0,14±0,07	0,4±0,2	0,30±0,15	0,16±0,14	0,008±0,01	0,09±0,05
4	0,8±0,5	0,3±0,2	0,06±0,03	0,2±0,18	0,17±0,08	0,08±0,05	0,004±0,01	0,07±0,11
5	1,2±0,6	0,4±0,2	0,09±0,05	0,46±0,3	0,24±0,1	0,11±0,05	0,004±0,004	0,03±0,02
6	1,3±0,6	0,4±0,2	0,1±0,1	0,27±0,1	0,18±0,06	0,15±0,14	0,008±0,01	0,13±0,06
7	2±0,4	0,9±0,1	0,09±0,06	0,76±0,2	0,2±0,14	0,21±0,05	0,004±0,004	0,1±0,07
8	1±0,4	0,4±0,2	0,07±0,03	0,37±0,14	0,16±0,06	0,1±0,02	0,003±0,002	0,07±0,05
9	1,4±0,5	0,5±0,2	0,07±0,06	0,47±0,29	0,17±0,12	0,1±0,05	0,010±0,005	0,02±0,01
10	0,8±0,3	0,3±0,1	0,06±0,02	0,16±0,18	0,17±0,07	0,08±0,03	0,003±0,004	0,09±0,15
11	1,5±0,3	0,4±0,1	0,06±0,02	0,47±0,27	0,15±0,04	0,13±0,05	0,008±0,01	0,06±0,06
12	1,8±0,5	0,7±0,2	0,06±0,02	0,54±0,2	0,14±0,05	0,1±0,02	0,009±0,01	0,03±0,03
13	1,3±0,3	0,2±0,1	0,07±0,02	0,36±0,1	0,16±0,04	0,08±0,02	0,005±0,005	0,02±0,01
14	2±0,8	0,6±0,3	0,08±0,02	0,59±0,3	0,18±0,05	0,15±0,06	0,004±0,005	0,01±0,01
15	1,2±0,6	0,4±0,2	0,08±0,05	0,46±0,4	0,18±0,1	0,11±0,05	0,004±0,005	0,05±0,05
