# Supplementary material for: Two-Year Study on the Intra-Individual Dynamics of Gut Microbiota and Short-Chain Fatty Acids Profiles in Healthy Adults
Source: Microorganisms. 2024 Aug 20;12(8):1712. doi: 10.3390/microorganisms12081712 (PMC11357285; doi:10.3390/microorganisms12081712)
Supplement: Supplementary file 1 [file microorganisms-12-01712-s001.zip › microorganisms-3144091-Table S4.pdf]

Supplementary Table 4. Seasonal changes in SCFA levels. Kruskal-Wallis test was used to calculate p adj values. p < 0.05 values were considered significant.

SCAFs	Season	M+-SD (mg/g)	p1 (Fall-Spring)	p2 (Fall-Summer)	p3 (Spring-Summer)	p4 (Fall-Winter)	p5 (Spring-Winter)	p6 (Summer-Winter)
C2 (Acetic)	Fall	1,19±0,6	0,88	0,28	0,35	0,57	0,30	1
	Winter	1,43±0,6						
	Spring	1,19±0,5						
	Summer	1,43±0,6						
C3 (Propionic)	Fall	0,38±0,2	0,35	0,2	0,75	0,2	0,97	0,79
	Winter	0,47±0,2						
	Spring	0,45±0,2						
	Summer	0,51±0,3						
iC4 (Isobutyric)	Fall	0,06±0,02	0,39	0,44	0,94	0,05	0,37	0,55
	Winter	0,09±0,05						
	Spring	0,09±0,09						
	Summer	0,07±0,03						
C4 (Butyric)	Fall	0,37±0,3	0,98	1	0,77	0,76	0,68	0,64
	Winter	0,42±0,3						
	Spring	0,36±0,2						
	Summer	0,46±0,3						
iC5 (Isovaleric)	Fall	0,14±0,04	0,26	0,3	0,91	**0,02**	0,5	0,37
	Winter	0,21±0,1						
	Spring	0,17±0,05						
	Summer	0,18±0,09						
C5 (Valeric)	Fall	**0,09±0,04**	0,15	**0,01**	0,30	0,16	0,84	0,13
	Winter	0,12±0,08						
	Spring	0,11±0,03						
	Summer	**0,14±0,09**						

**iC6 (Isocaproic)**	**Fall**	0,005±0,005	0,77000	0,66	0,88	0,8	0,65	0,46
	**Winter**	0,01±0,01						
	**Spring**	0,005±0,003						
	**Summer**	0,004±0,006						
**C6 (Caproic)**	**Fall**	1,15±0,05	0,99	1	0,92	0,83	0,74	0,87
	**Winter**	0,06±0,06						
	**Spring**	0,04±0,03						
	**Summer**	0,08±0,1						
